# Mapping integrated care for brain tumour–related epilepsy in the Italian RIN–IRCCS network

**DOI:** 10.1007/s10072-026-09188-w

**Published:** 2026-06-26

**Authors:** Elena Anghileri, Alessia Marcassoli, Veronica Villani, Giuseppe Didato, Claudia Milanaccio, Elena Pasini, Elisa Bennicelli, Pasquale Persico, Paola Bini, Giulia Berzero, Angela Mastronuzzi, Massimo Filippi, Luca De Palma, Giorgio Arcara, Anna Bellini, Marta Maschio, Andrea Pace, Mariangela Farinotti, Antonio Silvani

**Affiliations:** 1 Neuro-Oncology Unit, Fondazione IRCCS Istituto Neurologico Carlo Besta (FINCB), full member of the European Reference Network EURACAN, Milan, 20133 Italy; 2 Neurology, Public Health and Disability Unit, Fondazione IRCCS Istituto Neurologico Carlo Besta, Milan, 20133 Italy; 3 Neuro-Oncology Unit, IRCCS Regina Elena National Cancer Institute, Rome, 00144 Italy; 4 Epilepsy Unit, Fondazione IRCCS Istituto Neurologico Carlo Besta, full member of the European Reference Network EpiCARE, Milan, 20133 Italy; 5 Neuro-Oncology Unit, IRCCS Istituto Giannina Gaslini, Genoa, 16147 Italy; 6 Neurology Unit, IRCCS Istituto delle Scienze Neurologiche di Bologna, Bologna, 40139 Italy; 7 Medical Oncology Unit 2, IRCCS Ospedale Policlinico San Martino, Genoa, 16132 Italy; 8 Hematology and Medical Oncology Unit, IRCCS Humanitas Research Hospital, Rozzano, 20089 Italy; 9 Neurooncology Unit, IRCCS Mondino Foundation, Pavia, 27100 Italy; 10 Neurology Unit, IRCCS Ospedale San Raffaele, Milan, 20132 Italy; 11 Neuro-Oncology Unit, Bambino Gesù Children’s Hospital, IRCCS, Rome, 00165 Italy; 12 Neuroimaging Research UnitNeurology UnitNeurorehabilitation Unit, and Neurophysiology Service, IRCCS Ospedale San Raffaele, Vita-Salute San Raffaele University, Milan, 20132 Italy; 13 Neurology, Epilepsy and Movement Disorders Unit, Bambino Gesù Children’s Hospital, IRCCS, Full Member of European Reference Network on Rare and Complex Epilepsies, EpiCARE, Rome, 00165 Italy; 14 IRCCS San Camillo Hospital, Venice, Italy; 15 Department of General Psychology, University of Padua, Padua, Italy; 16 Neurology Unit and Neurophysiology Service - IRCCS Ospedale San Raffaele, Milan, 20132 Italy

**Keywords:** Brain tumour-related epilepsy, Brain tumour, Epilepsy, Neuro-oncology, Multidisciplinary care, Clinical pathways

## Abstract

**Background:**

Brain tumour-related epilepsy is a frequent and disabling complication of primary and metastatic brain tumours, requiring multidimensional and coordinated management. However, specific guidelines and standardized care pathways for this condition are still lacking. This study aimed to assess the current organization of care for brain tumour-related epilepsy within the Italian Network of Neuroscience and Neuro-rehabilitation Research Institutes.

**Methods:**

A national, multicentre survey was conducted across participating centres between April and June 2024, with follow-up validation in December 2025. The questionnaire investigated human resources, technological availability, clinical pathways, and management strategies. Data were analysed descriptively.

**Results:**

Ten centres participated. All reported adopting a multidisciplinary approach, although with marked variability in team composition, expertise, and resources. Core specialists were available in most centres, while paediatric expertise was often lacking. Advanced neurophysiological monitoring and antiseizure medication level assessment were not uniformly accessible. Magnetic Resonance imaging was widely available, whereas Positron Emission Tomography was less consistently implemented. No centre had a dedicated clinical pathway for brain tumour–related epilepsy, despite the presence of pathways for brain tumours and epilepsy separately. Neuropsychological and comorbidity assessments were inconsistently integrated into care.

**Conclusions:**

These findings highlight significant heterogeneity in the management of brain tumour-related epilepsy, even in highly specialized settings. The development of dedicated, standardized clinical pathways is needed to harmonize care and improve patient outcomes.

**Supplementary Information:**

The online version contains supplementary material available at https://doi.org/10.1007/s10072-026-09188-w.

## Introduction

In 2023, the Italian Ministry of Health launched the National Virtual Institute of Neuro-Oncology network, within the broader framework of RIN (Neurological Network of Neuroscience and Neurorehabilitation of the Italian Scientific Research and Clinical Care Institutes (IRCCS)) [1]. RIN is a national network focused on neuroscience and involves all IRCCS institutions with the aim of developing research programmes for the treatment of neuro-oncological conditions and the neurological complications of cancer therapies [2].

The RIN-IRCCS network proposed several thematic areas to be developed and integrated among specialists. Within the neuro-oncology thematic group, the most prominent, yet previously underexplored issue identified was brain tumour-related epilepsy (BTE). The RIN Network represents 92% (11/12) of all the Italian IRCCS institutions and 85% (11/13) of all the Italian Hospitals with a neuro-oncology unit.

According to the International League Against Epilepsy (ILAE), BTE is an aetiology- specific epilepsy syndrome secondary to primary or metastatic brain tumours; BTE poses significant challenges in terms of treatment and prognosis [3]. Among primary brain tumours, the incidence of epilepsy varies by tumour type, with low-grade gliomas associated with a higher risk compared to high-grade gliomas [4], although at least one seizure occurs in up to 80% of patients with high-grade glioma during the course of the disease [5].

Primary tumours of the central nervous system (CNS) have an incidence rate of 5 cases per 100,000 individuals per year in Europe and account for approximately 2% of all cancer-related deaths [6, 7]. Over recent decades, there has been a gradual but steady increase in incidence, particularly of high-grade gliomas among individuals over the age of 65 [8]. In this already frail population, the presence of epilepsy further compromises the clinical picture.

BTE significantly affects multiple domains, including physical impairment, neurocognitive disturbances, and side effects related to antiseizure medications. Additionally, lifestyle changes and challenges within the social and family context may lead to temporary or even permanent dependency.

In patients with brain tumour, tumour-related symptoms and epilepsy require distinct yet compatible treatment strategies to avoid an increased risk of adverse effects and drug-drug interactions, particularly between antiseizure medications and antitumor drugs or radiation therapy [9]. Given the complexity of this dual pathology, a multidisciplinary approach is strongly recommended [10], as it has been shown to improve outcomes for these patients [11, 12]. However, the implementation of such multidisciplinary models remains heterogeneous across European healthcare systems [12], including within Italy.

In 2006, the National Institute for Health and Care Excellence (NICE) published the guideline “*Improving Outcomes for People with Brain and Other CNS Tumours* recommending that care be delivered through multidisciplinary team (MDT), comprising neurologists, oncologists, and other supporting healthcare professionals (HCPs) [13]. These recommendations have shaped international standards increasing the role of neurologists in neuro-oncology although training in this field remains limited in most medical schools and residency programs [14].

The Italian Ministry of Health updated the National Guidelines for the management of brain tumours in 2023 aiming to standardize care and provide an evidence-based reference for institutions; however, they do not specifically address the clinical setting of BTE patients [15].

Guidelines from the Society of Neuro-Oncology (SNO) for BTE are not yet available, and their consensus review on current management has identified electroencephalography (EEG) as a tool to aid in the diagnosis of BTE [9]. The European Association of Neuro-Oncology (EANO), guidelines mainly focus on seizure prophylaxis in patients with brain tumours [16] and on recommendations for managing treatment-related complications offering limited guidance on BTE management beyond the perioperative phase [17]. An Italian multidisciplinary paper has summarized key evidence and therapeutic advancements for BTE [18]. Guidelines are essential for the development of local clinical pathways (CPs), which are official documents approved by the competent bodies of each hospital.

To understand the current state of care for BTE patients in Italy, and to identify sources and extent of variability in care while assessing the modifiability of contributing factor, we promoted a survey among RIN centres.

## Materials and methods

We developed a multicentre, national survey within the RIN network to investigate how BTE patients are managed. We adapted to our scope a questionnaire previously developed and validated by the Italian Epilepsy Surgery Collaborative Group [19]. The survey was conducted online between April and June 2024, and participating centres were re-contacted in December 2025 to confirm the collected data and assess whether any changes in clinical practice had occurred during the intervening period.

A specialist — such as a general neurologist, epileptologist, neuro-oncologist, neurosurgeon, oncologist, or psychologist — who was actively involved in the clinical management of BTE and formally delegated by the Institution’s scientific coordinator, was invited to complete the questionnaire. Responses were compiled in collaboration with the hospital’s administrative data centre to ensure accuracy of institutional data.

Ethical approval to carry out the survey was given by the RIN network committee prior to data collection. Additionally, all methods were performed in accordance with the relevant guidelines, standard operating procedures and regulations. All procedures were conducted in accordance with the Declaration of Helsinki.

The online survey consisted predominantly of closed-ended items (dichotomous or multiple-choice), rating-scale items, and a limited number of open-ended questions. The questionnaire consisted of eight sections designed to gather data on the management of patients with BTE. The eight sections were: (1) human resources; (2) infrastructural and technological resources; (3) clinical trial activity; (4) diagnostic–therapeutic CPs; (5) participation in clinical networks; (6) tumour pathology registries; (7) management of individuals with BTE; and (8) comorbidity assessment. Some sections included specific questions also for brain tumours, and epilepsy. The term CP referred to an official document approved by each hospital. (For full questionnaire, see Supplementary Material S1). Non-responding centres were re-contacted up to five times.

### Statistics

All variables were analysed using descriptive statistics and a qualitative approach. Background information on respondents and centres was reported descriptively. Descriptive statistics were presented as means and ranges for continuous variables, and as absolute numbers (n), frequencies, and percentages for categorical variables. Qualitative variables were summarised in terms of absolute frequency and percentage. Data analysis was performed using Microsoft Excel.

## Results

Ten out of the eleven RIN hospitals completed the questionnaire; response rate was 100% for all the survey items. Responses were provided by different specialists: predominantly neuro-oncologist (*n* = 4), and epileptologist (*n* = 3), general neurologist (*n* = 1), oncologist (*n* = 1), and psychologist (*n* = 1).

**Fig. 1:**
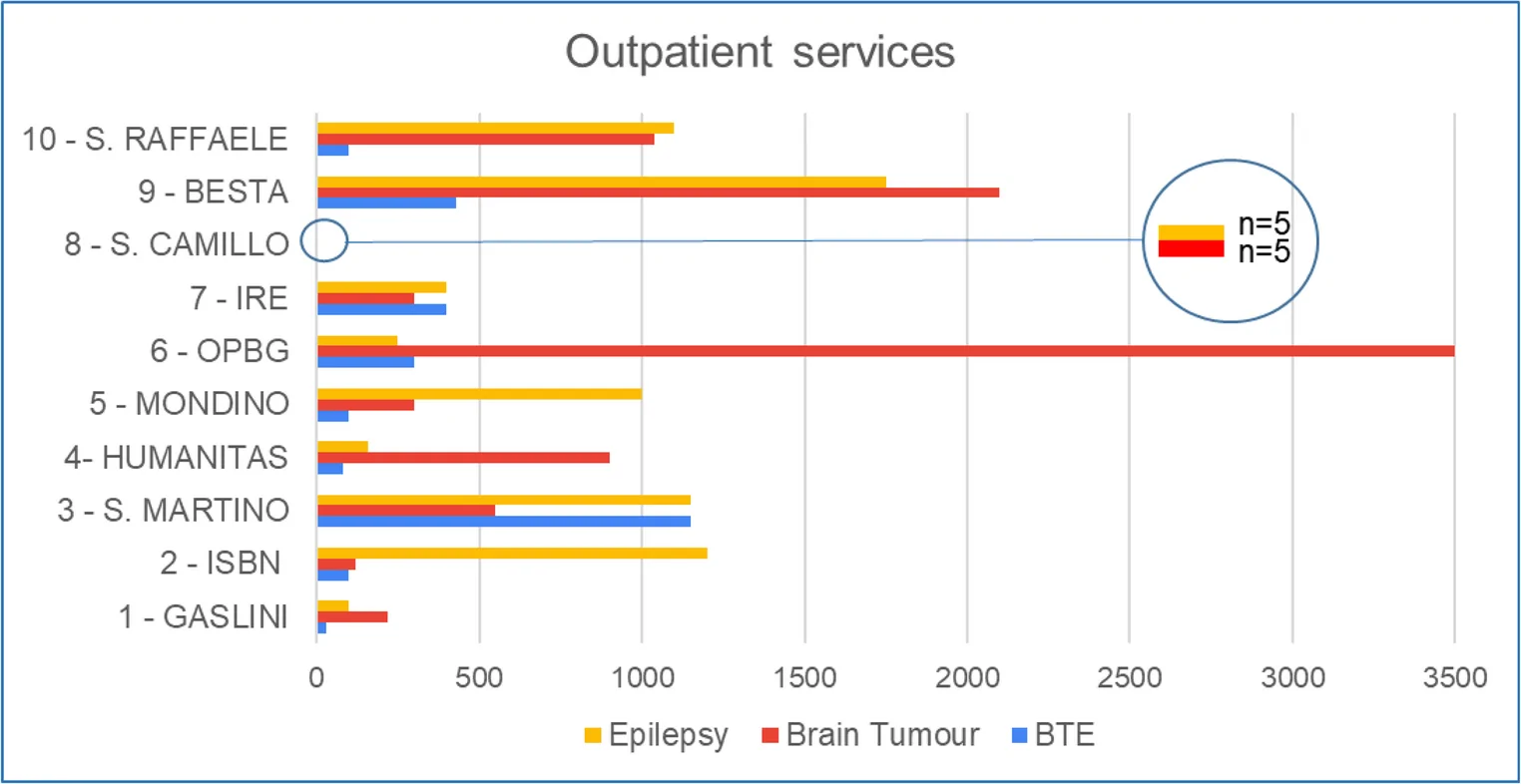
Number of patients attending outpatient services across the ten participating IRCCS centres in 2023. Legend: Centre number: 1: IRCCS Istituto Giannina Gaslini, Genoa; 2: IRCCS Istituto delle Scienze Neurologiche, Bologna; 3: IRCCS Ospedale Policlinico San Martino, Genoa; 4: IRCCS Humanitas, Milan; 5: IRCCS Fondazione C. Mondino, Pavia; 6: IRCCS Bambino Gesù Children’s Hospital, Rome; 7: IRCCS Regina Elena, Rome; 8: IRCCS San Camillo s.r.l., Venice; 9: Fondazione IRCCS Istituto Neurologico Carlo Besta, Milan; 10: IRCCS Ospedale San Raffaele, Milan

The ten participating hospitals reported heterogeneous volumes of both inpatients and outpatients with BTE. In particular, the number of inpatient beds for BTE ranged from 6 to 38. A similar variability was observed in the numbers for brain tumour and for epilepsy (Fig. 1).

### Human resources

To determine whether each centre had the HCPs required to build an MDT dedicated to BTE, we asked which specialists were involved in the management of brain tumours and which were involved in the management of epilepsy, respectively. Results are presented in Tables 1 and 2.

**Table 1:** Dedicated health board

EPILEPSY	0 HCPs	1–2 HCPs	3–5 HCPs	> 5 HCPs
Neurologist	0	3	3	4
Neurophysiologist	3	3	0	4
Child neurologist	6	0	3	1
Paediatrician	7	2	0	1
Psychologist	2	5	1	2
Neuroradiologist	1	4	2	3
Neurosurgeon	3	3	3	1
Nutritionist	3	5	2	0
Physiotherapist/ speech therapist	3	3	1	3
Technician of Neurophysiology	1	3	2	4
Dedicated nurse or socio-medical worker	2	1	3	4
Social worker	5	3	2	0
Other dedicated health personnel	3	5	1	1
BRAIN TUMOR	0 HCPs	1–2 HCPs	3–5 HCPs	> 5 HCPs
Neurologist	1	2	5	2
Neurophysiologist	4	4	2	0
Child neurologist	7	2	1	0
Paediatrician	8	1	0	1
Psychologist	1	5	3	1
Neuroradiologist	0	4	3	3
Neurosurgeon	2	1	6	1
Radiotherapist	3	4	3	0
Oncologist	1	5	3	1
Palliativist	4	5	1	0
Nutritionist	4	6	0	0
Physiotherapist/ speech therapist	0	6	3	1
Technician of Neurophysiology	1	5	4	0
Dedicated nurse or socio-medical worker	1	4	2	3
Social worker	4	5	1	0
Other dedicated health personnel	3	6	1	0

Specialists/Health care professionals (HCPs) (number) dedicated to epilepsy and brain tumour in the RIN network

**Table 2:** Instrumental exams for BTE patients

	Yes, presence of (N)	%
Neurophysiology Techniques		
Standard Electroencephalography (EEG)	10	100
Daytime Polysomnography	7	70
Night Polysomnography	5	50
Video-EEG	6	60
Long-term monitoring	7	70
High-density EEG	7	70
Advanced techniques (EEG source imaging, back averaging, other)	7	70
EEG-functional Magnetic Resonance	2	20
Magnetoencephalography	1	10
Multimodal Evoked Potential (EP)		
Motor EP	9	90
Somatosensory EP	9	90
Brainstem Auditory EP	9	90
Visual EP	8	80
Intraoperative neurophysiological monitoring	7	70
Invasive neurophysiology	3	30
Imaging		
Brain Computer Tomography	9	90
Brain Magnetic Resonance 1.5T	8	80
Brain Magnetic Resonance 3T	9	90
Brain Magnetic Resonance 7T	0	0
Brain PET (any tracer)	6	60
PET FDG	6	60
PET MET	3	30
Brain functional Magnetic Resonance	9	90
Speech	9	90
Motor	8	80
Memory	5	50
Morphometric techniques (post-processing)	8	80
Blood exams		
Plasma Anti-Seizure Medication level	7	70

Technologies to evaluate BTE patients in the RIN network

All centres reported the availability of dedicated personnel; however, differences in expertise were observed across centres. Some centres showed a high level of specialisation in paediatric neuro-oncology, whereas this expertise is generally absent in non- specialised settings. For instance, 60% and 70% of centres reported no child neurologist dedicated to epilepsy and brain tumours, respectively, and 70% and 80% lacked a paediatrician for the same conditions. Full details are provided in Tables 1 and 2.

The number of patients accessing outpatient services varied significantly across centres, with some hospitals showing a predominance of either brain tumour cases or epilepsy cases (Fig. 1).

**Fig. 2:**
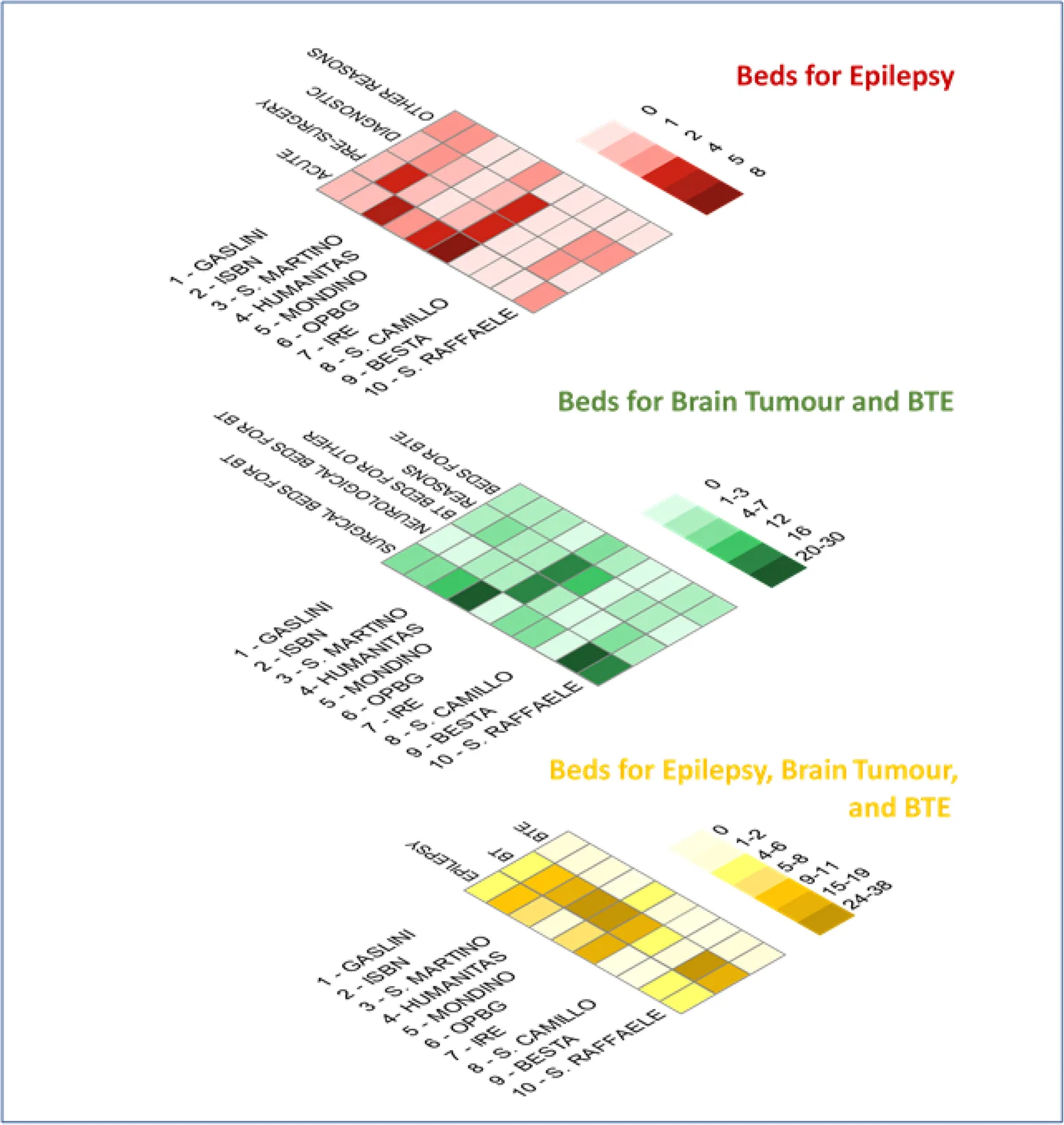
Number of inpatient beds allocated for brain tumour epilepsy (BTE), brain tumours, and epilepsy across the ten participating IRCCS centres in 2023. Legend: Centre number: 1: IRCCS Istituto Giannina Gaslini, Genoa; 2: IRCCS Istituto delle Scienze Neurologiche, Bologna; 3: IRCCS Ospedale Policlinico San Martino, Genoa; 4: IRCCS Humanitas, Milan; 5: IRCCS Fondazione C. Mondino, Pavia; 6: IRCCS Bambino Gesù Children’s Hospital, Rome; 7: IRCCS Regina Elena, Rome; 8: IRCCS San Camillo s.r.l., Venice; 9: Fondazione IRCCS Istituto Neurologico Carlo Besta, Milan; 10: IRCCS Ospedale San Raffaele, Milan

Inpatient service workloads for BTE, brain tumour, and for epilepsy are summarised in Fig. 2, with values ranging from very few cases to several thousand across centres. For full questionnaire details, see Supplementary Material S1.

### Infrastructural and technological resources

We investigated the technological facilities available for the management of BTE across the ten participating centres, including neurophysiology, imaging, clinical software, and plasma antiseizure medication level detection. The results are summarised in Table 2. In particular, plasma antiseizure medication assays and extensive neurophysiological monitoring (including video-EEG, long-term monitoring, and intraoperative monitoring) were available in only 70% of centres. Brain Magnetic Resonance (MR) imaging was reported in 90% of the centres; brain Positron Emission Tomography (PET) imaging was available in 60% of centres, with variability in the tracers used.

### The clinical pathways

Specific diagnostic and therapeutic CPs for BTE were entirely lacking across the surveyed centres. In contrast, 80% of centres had established CPs for brain tumours, and 70% had CPs for epilepsy.

All centres reported a multidisciplinary approach to BTE management, although organisational models were heterogeneous. Across all centres, patients with brain tumours at risk of epilepsy underwent regular consultations with either an epileptologist or a neuro-oncologist. EEG was routinely used in 70% of centres.

Neuropsychological assessment was included in standard care in 40% of centres, while psychological support was available in 90% of centres and extended to caregivers in 60%. Psychiatric evaluations were performed in 70% of centres, with specific psychiatric testing reported in 71.4% (5/7) of these cases.

Comorbidities such as sleep disorders, nutritional issues, and fertility-related concerns were specifically addressed in 70%, 90%, and 80% of centres, respectively. In addition, dedicated assessment tools were used for the evaluation of sleep disorders in 71.4% (5/7) of cases and for nutritional assessment in 22% (2/9).

### Additional information

Across centres, pathologists processed an annual mean of 250 primary brain tumour samples (range 0–900), including both newly diagnosed tumours and surgical specimens from relapsed cases.

Molecular-genetic testing was widely available including Sanger sequencing (*n* = 4), digital PCR (*n* = 5), next-generation sequencing (NGS) (*n* = 6), multiplex ligation-dependent probe amplification (MLPA) (*n* = 2), and methylome analysis (*n* = 3).

All centres also served as clinical trial sites. Specifically, 70% had a dedicated team comprising physicians, data managers/study coordinators, and study nurses; 10% had only physicians and data managers/study coordinators; while 20% had exclusively data managers/study coordinators.

80% and 70% of centres were involved in national and international epilepsy networks, respectively, while 100% and 80% participated in national and international BT networks.

Tumour pathology registries were present in 90% of the centres.

All 2023 data were confirmed by the hospitals in December 2025 and no institutions reported significant changes in their organizational or clinical approach to BTE patients.

## Discussion

National and international guidelines for the management of BTE are currently lacking [2, 20]. In this study, we assessed the variability of care for BTE patients within neuro-oncological centres of the Italian RIN network and documented differences in both dedicated personnel and available technology.

The use of CPs has been shown to enhance quality of care, with patients managed within such pathways experiencing lower excess mortality across cancer types [21]. Compared to usual care, CPs are consistently associated with improvements in quality of care, service efficiency, and interdisciplinary collaboration [22].

An appropriate CP requires an MDT and access to complementary instrumental examinations [20].

As summarized in Tables 1 and 2, some HCPs and technologies are pivotal for BTE management.

Based on expert opinion within the RIN network, a minimum set of core specialists for BTE management should include the presence of neurologist, oncologist or neuro-oncologist, neurosurgeon, epileptologist, neuropsychologist. This minimum request is fulfilled by 70% of centres in the RIN network. Other specializations, e.g., radiotherapist, child neurologist and neuro-radiologist, are context-specific depending on tumour histology, patient’s age, and hospital specialization. Some roles (e.g. nutritionist) play a supportive function.

Similarly, certain technologies should be considered mandatory (e.g. EEG, at least 1.5-Tesla brain MR, plasma antiseizure levels), while others are complementary (e.g. EEG-fMR, magnetoencephalography).

The current survey identified potential areas for improvement: dedicated CNS tumour neuropathologist, plasma antiseizure medication assays, polysomnography, and extensive intraoperative neurophysiological monitoring -all of which were available in less than 80% of centres- should represent priority targets for care recommendations. In addition, a broader use of brain PET imaging could be beneficial, especially if recent findings supporting its role in the pre-operative assessment of low-grade gliomas are confirmed [23].

As most RIN-IRCCS centres are specialized tertiary care centres and participate in national and international research networks dedicated to epilepsy and brain tumour, our findings likely reflect the current best practices in Italy.

Our work is situated within an evolving international context that increasingly recognises the need for tailored CPs in neuro-oncology [24]. Notably, CPs specific to brain tumour have been reported in fewer than one-third of European institutions [15].

The main limitations of the study are related to the survey design aimed at collecting descriptive data. The investigation relied on data provided by the participating centres, which were consistently asked to cross-check their responses with administrative sources. Differences in the professional backgrounds of respondents may also have influenced the results; however, all respondents were members of their hospitals’ neuro-oncology teams and had direct knowledge of the organisational and multidisciplinary networks. Quantitative data were cross-checked against administrative sources. Although the response rate was high (10 out of 11 centres), the sample size remains limited, precluding stratified analyses or comparisons. The cross-sectional nature of the survey provides only a snapshot of the current situation, without capturing temporal changes. Furthermore, the survey design - which focused primarily on organisational and structural aspects of BTE management - did not allow us to analyse or determine associations between resources/structures and process indicators or short and long-term outcomes.

Despite these limitations, the present study contributes to defining baseline standards for implementation of standardized care practice targeting BTE.

This real-world survey provides an initial framework and represents a first step toward bridging the gap between real-world practice and evidence-based care. The framework will involve analysing and integrating data to identify unmet needs, practice variations, and management gaps, followed by a multidisciplinary Delphi consensus including specialists and patient representatives.

### Implication for practice

The observed variability in specialist availability and diagnostic resources indicates unequal access to comprehensive care and the need to implement standardized, multidisciplinary care pathways for BTE. Establishing minimum organisational and technological standards could improve care coordination and promote more consistent patient management.

A concise set of recommendations for the management of BTE, with practical implications for everyday neuro-oncological practice, is provided in the following box.

Box. Practical Implications for Brain Tumour Epilepsy (BTE) Management Domain

**Table:** 

	Minimum standard / Recommendation	Expected impact
Care organisation	Implement standardized multidisciplinary care pathways (CPs) for BTE.	Reduce variability and improve equity of care.
Core multidisciplinary team (MDT)	Neurologist, neuro-oncologist/oncologist, neurosurgeon, epileptologist.	Improve coordinated and comprehensive management in MDT.
Essential diagnostics	EEG, ≥ 1.5-T brain MRI, plasma antiseizure medication level detection.	Support timely diagnosis and treatment optimisation.
Priority areas for improvement	Dedicated neuropathology, plasma antiseizure drug level detection, larger integrated MDT.	Strengthen diagnostic accuracy and management care.
Future implementation	Develop harmonised national and European recommendations and adaptable CPs.	Promote consistent, high-quality BTE management across centres.

Abbreviations: Brain Magnetic Resonance imaging (MRI); electroencephalography (EEG), multidisciplinary team (MDT)

## Conclusion

This survey reveals substantial and actionable variability in the organisation and resources dedicated to the management of BTE across the RIN network, reflecting the absence of dedicated guidelines. Although centres exhibit strong adherence to current best practices, critical disparities in expertise, and essential diagnostics and technological resources persist.

These findings underscore the need for European and national guidelines, and their translation into harmonised CP tailored specifically for BTE. Implementing such pathways would represent a transformative step toward consistent, high-quality, and equitable care across neuro-oncological settings, narrowing current gaps and improving patient outcomes.

## Supplementary Information

Below is the link to the electronic supplementary material.

Supplementary Material 1

## Data Availability

Data will be available at: https://zenodo.org/communities/besta/. (10.5281/zenodo.15056694)
