# Supplementary material for: Mapping integrated care for brain tumour–related epilepsy in the Italian RIN–IRCCS network
Source: Neurol Sci. 2026 Jun 26;47(7):589. doi: 10.1007/s10072-026-09188-w (PMC13303563; doi:10.1007/s10072-026-09188-w)
Supplement: Supplementary file 1 — Supplementary Material 1 [file 10072_2026_9188_MOESM1_ESM.docx]

**Patients with tumour-related epilepsy and their health professionals’ management: lessons to learn**

**SURVEY**

**Tumour Epilepsy: Tumour epilepsy survey in the RIN network**

*The survey is aimed at assessing*

*a. human resources dedicated to epilepsy and brain tumours*

*b. infrastructural and technological resources dedicated to epilepsy, brain tumours and specifically to tumour epilepsy;*

*c. clinical trial centres;*

*d. PDTA by pathology;*

*e. networks;*

*f. pathology registers;*

*g. management of patients with cancer epilepsy;*

*h. comorbidity assessment.*

**a. Human resources**

**a1. Human resources dedicated to epilepsy**

The questions in this section will investigate the number of staff for each specialised fi gure, including both staff on staff and fl exible contracts (to be indicated as a percentage in the last question)

1.

Number of neurologists: 0; 1-2; 3-5; >5

2.

Numbers of neurophysiologists: 0; 1-2; 3-5; >5

3.

Number of child neurologists: 0; 1-2; 3-5; >5

4.

Number of pediatricians: 0; 1-2; 3-5; >5

5.

Number of psychologists: 0; 1-2; 3-5; >5

6.

Number of neuro-radiologists: 0; 1-2; 3-5; >5

7.

Number of neuro-surgeons: 0; 1-2; 3-5; >5

8.

Number of nutritionists: 0; 1-2; 3-5; >5

9.

Number of rehabilitators: 0; 1-2; 3-5; >5

10.

Number of neurophysiology technicians: 0; 1-2; 3-5; >5

11.

Number of dedicated nurses or socio-medical operator: 0; 1-2; 3-5; >5

12.

Number of social workers: 0; 1-2; 3-5; >5

13.

Number of other dedicated health personnel: gynaecologist/obstetrician; geneticist; nuclear radiologist; other; none

14.

What percentage of the professional figures above represent permanent staff?

<10%; 10-24%; 25-49%,; 50-74%; > 75%

**a2. Human resources dedicated to primary brain tumours**

The questions in this section will investigate the number of staff for each specialised figure (including both staff on staff and flexible contracts, to be indicated as a percentage in the last question of this section)

1.

Number of neurologists: 0; 1-2; 3-5; >5

2.

Numbers of neurophysiologists: 0; 1-2; 3-5; >5

3.

Number of child neurologists: 0; 1-2; 3-5; >5

4.

Number of pediatricians: 0; 1-2; 3-5; >5

5.

Number of psychologists: 0; 1-2; 3-5; >5

6.

Number of neuro-radiologists: 0; 1-2; 3-5; >5

7.

Number of neuro-surgeons: 0; 1-2; 3-5; >5

8.

Numbers of radiotherapists: 0; 1-2; 3-5; >5

9.

Numbers of oncologists: 0; 1-2; 3-5; >5

10.

Numbers of palliatives: 0; 1-2; 3-5; >5

11.

Number of nutritionists: 0; 1-2; 3-5; >5

12.

Number of rehabilitators: 0; 1-2; 3-5; >5

13.

Number of neurophysiology technicians: 0; 1-2; 3-5; >5

14.

Number of dedicated nurses or socio-medical operator: 0; 1-2; 3-5; >5

15.

Number of social workers: 0; 1-2; 3-5; >5

16.

Number of other dedicated health personnel: gynaecologist/obstetrician; geneticist; nuclear radiologist; other; none

17.

What percentage of the professional figures above represent permanent staff?

<10%; 10-24%; 25-49%,; 50-74%; > 75%

**b. Infrastructural and technological resources**

**b1.**

**Infrastructure resources dedicated to epilepsy**

1.

Epilepsy outpatient visits/year (Reference year 2023)

Number of beds dedicated to epilepsy FOR ACUTE PATIENTS

(if you do not have dedicated beds, please indicate how many beds on average are occupied by patients for epilepsy management IN ACUTE: e.g.: 20 beds in your OU of which 5 on average are occupied by patients with epilepsy)

2.

Number of beds dedicated to epilepsy PER PRE-SURGERY STUDY:

(if you do not have dedicated beds, please indicate how many beds on average are occupied by patients PER PRE-SURGICAL STUDY for epilepsy: e.g.: 20 beds in your OU of which 5 on average are occupied by pts with epilepsy)

3.

Number of beds dedicated to epilepsy PER DIAGNOSTIC STUDY:

(if you do not have dedicated beds, please indicate how many beds on average are occupied by people with epilepsy PER DIAGNOSTIC STUDY: e.g. 20 beds in your OU of which 5 on average are occupied by people with epilepsy)

4.

Number of beds dedicated to epilepsy FOR CAUSES OTHER THAN THESE, i.e. not ‘for acute’, nor ‘for pre-surgery study’, nor ‘per diagnosis’

(if you have no dedicated beds, please indicate how many beds on average are occupied by people with epilepsy: e.g. 20 beds in your unit of which 5 on average are occupied by people with epilepsy)

**b2.1**

**Dedicated infrastructure resources for brain tumours (BT)**

1.

Outpatient visits for BT/year (Reference year 2023)

2.

Number of dedicated SURGICAL beds for BT

*(if there are no dedicated beds, please indicate how many beds on average are occupied by patients with BT: e.g. 20 beds in your unit of which 5 beds on average are occupied by patients with BT)*

3.

Number of NEUROLOGICAL beds dedicated to BT

*(if there are no dedicated beds, please indicate how many beds on average are occupied by people with BT: e.g. 20 beds in your unit, of which 5 beds on average are occupied by people with BT)*

4.

Number of beds dedicated to brain tumours for reasons DIFFERENT from surgical and neurological ones

*(if you have no dedicated beds, please indicate how many beds on average are occupied by people with BT: e.g. 20 beds in your CU of which 5 on average are occupied by people with BT)*

**b2.2**

**Dedicated infrastructure resources for BTE**

*

Epilepsy-specific outpatient visits in brain tumours/year (Reference year 2023;

if no service exists: enter ‘0”

Number of beds dedicated to the study of Tumour Epilepsy

(if there are no dedicated beds, please indicate how many beds on average are occupied by patients with epilepsy: e.g. 20 beds in your operating unit of which 5 on average are occupied by patients with epilepsy)

**b3. Dedicated epilepsy diagnostic investigations - Neurophysiology**

Evaluation of neurophysiology technologies available for the study of tumour epilepsy

1.

Standard EEG: yes; no

2.

Daily polysomnography: yes; no

3.

Nocturnal polysomnography: yes; no

4.

Video EEG: yes; no

5.

Long-term monitoring (LTM): yes; no

6.

High-density EEG (HD-EEG): *yes; no*

7.

Advanced EEG as EEG *source imaging EEG (ESI), back averaging, others: yes; no*

*8.*

*EEG-functional MR (fMR): yes; no*

*9.*

*Magnetoencephalography: yes; no*

10.

Multimodal evoked potential:

sensory, motor, visual, brainstem, none

11.

Intraoperative neurophysiological monitoring: Phase Reversal; Mapping with DCS (direct cortical stimulation); Cortico-cortical potentials; Electrocorticography (ECoG); Awake surgery; None

12.

Invasive neurophysiological assessment: Oval Foramen Electrodes; Subdural Electrodes; Stereo-EEG; none

**b4. Evaluation of imaging machineries** (available within the Institute or through specific agreements)

evaluation of neuroimaging technologies available for the study of BTE

1.

Brain Computer Tomography (CT): yes; no

2.

Brain Magnetic Resonance (MR) at 1.5 T: yes; no

3.

Brain Magnetic Resonance (MR) at 3 T: yes; no

4.

Brain Magnetic Resonance (MR) at 7 T: yes; no

5.

Brain Positron Emission Tomography (PET): yes, with FDG tracer; yes with amino acid tracer; yes, with both FDG and amino acid tracer; none

6.

fMR: yes, for motor study; yes, for language study; yes, for memory study; yes, for motor and language study; yes, for language and memory study; yes, for motor and memory studies; yes, for motor, language and memory study; yes, for other study; no

7.

Morphometry techniques (post-processing); yes; no

**b4.**

**Tumour Epilepsy Diagnostic Investigations - Laboratory Analyses**

Includes General Laboratory Analysis, Pathological Anatomy, Molecular Genetics Laboratory

1.

Plasma level measurement of Anti-Seizure Medication (ASM): yes, no

2.

Dedicated anatomo-pathology for BT diagnosis.

Indicate number of central nervous system (CNS) tumour samples (Reference year 2023)

If Dedicated anatomo-pathology is ABSENT: enter ‘0’

3.

Molecular and genetic Laboratory

Indicate at least 1 or more answers: Sanger; Digital PCR; Next Generation Sequencing (NGS); [Multiplex ligation-dependent probe amplification](https://www.igenomix.it/genomics-precision-diagnostic/single-gene-analysis/mlpa/) (MLPA); Methylome profiling; other; none

**c.**

**Clinical trial center**

Evaluate if the Institute is involved in clinical trial

Is the institute involved in clinical trials? yes; no

If yes:

-the Institute is involved as: promoter and participant; only as participant; in neuro-oncological setting; in epilepsy context

-are health personnel specifically involved in clinical trial? (check also more than one answer) no; medical doctors; data manager/study coordinators; study nurse

**d.**

**Diagnostic therapeutical care path**

1.

Are there diagnostic therapeutical care paths for epileptic patients? yes; no

2.

Are there diagnostic therapeutical care paths for patients with primary BT? yes; no

3.

Are there diagnostic therapeutical care paths for patients with BTE? yes; no

**e.**

**Diseases networks**

1.

Is the Institute involved in national epileptic network? yes; no

2.

Is the Institute involved in international epileptic network? yes; no

3.

Is the Institute involved in national BT network? yes; no

4.

Is the Institute involved in international BT network? yes; no

**f.**

**Disease registry**

1.

Does the Institute a registry dedicated to epilepsy and related diseases? yes; no

2.

Does the Institute a registry dedicated to BT? yes; no

**g.**

**Management of patients with BTE**

1.

The patient with BTE is usually managed by: internal neuro-oncologist; facility neurologist in consultation with oncologist; internal epileptologist in consultation with neuro-oncologist; facility epileptologist in consultation with neuro-oncologist ONLY IF drug resistant epilepsy occurs; in institutes other than the main affereting for the two pathologies (BT and epilepsy); other

2.

The newly diagnosed BT with epileptogenic potential patient: is always subjected to EEG; always undergoes neuropsychological testing; is always subjected to specific epileptological/neurological assessment; none of the above

3.

The subject with BTE has the possibility of receiving targeted psychological support therapy (including group therapy): yes, in the Institute; yes, but on satellite Institute; no

4.

The caregiver of BTE patient has the possibility of receiving targeted psychological support therapy (including group therapy): yes, in the Institute; yes, but on satellite Institute; no

5.

The subject with BTE is periodically (at least once/year) subjected to: EEG; neuropsychological tests; quality of life (QoL) test; none

**f.**

**Clinical and instrumental assessment of comorbidities and gender issues**

1.

Cognitive disorders are evaluated by: clinical evaluation only; clinical assessment and neuropsychological testing (specify which); none

2.

Psychiatric profiling is evaluated by: clinical evaluation only; clinical assessment and neuropsychological testing (specify which); none

3.

Sleeping disorders are evaluated by: clinical evaluation only; clinical assessment and neuropsychological testing (specify which); none

4.

Nutritional disorders are evaluated by: clinical evaluation only; clinical assessment and neuropsychological testing (specify which); none

5.

Fertility/fecondation/pregnancy issues are evaluated by: clinical evaluation only; clinical and instrumental assessment; none
